# Effect of IL-6 inhibition on lipoprotein(a) levels: A systematic review and meta-analysis

**DOI:** 10.1016/j.ajpc.2026.101498

**Published:** 2026-02-22

**Authors:** Saeid Mirzai, James McParland, Muhammad Imtiaz Ahmad, Emil deGoma, John Walsh, Michael D. Shapiro

**Affiliations:** a Center for Prevention of Cardiovascular Disease, Department of Cardiovascular Medicine, Wake Forest University School of Medicine, Winston-Salem, NC, USA; b Department of Internal Medicine, Section on Hospital Medicine, Medical College of Wisconsin, Milwaukee, WI, USA; c Tourmaline Bio, Inc., NY, USA

**Keywords:** Interleukin-6, Lipoprotein(a), Monoclonal antibody, Anti-IL-6, Anti-IL-6R, Cardiovascular risk

## Abstract

**Background:**

Inhibition of interleukin (IL)-6 signaling by monoclonal antibodies (mAb) inhibits apolipoprotein(a) synthesis and reduces circulating lipoprotein(a) [Lp(a)] levels.

**Aims:**

The aims of this systematic literature review and meta-analysis were to summarize clinical evidence for and quantify the effect of anti-IL­-6/IL-6 receptor (IL-6R) treatment on Lp(a) levels. Secondary inflammatory and lipid endpoints were also examined.

**Methods:**

Searches were conducted using Embase, PubMed and Cochrane Library databases to identify studies reporting changes in Lp(a) levels following repeat doses of anti-IL-6/IL-6R mAbs. A random-effects meta-analysis compared the effect of IL-6 inhibition vs comparator on Lp(a) levels. A pre­-post analysis assessed the effect of IL-6 inhibition on Lp(a) levels before and after treatment. Subgroup analyses by disease type, IL-6 inhibition agent, and comparators were conducted.

**Results:**

Out of 96 records, 10 studies with 1,201 participants were included. IL-6 inhibition was associated with significant reductions in Lp(a) levels vs baseline at 2–3 months (standardized mean difference [SMD] -0.29; 95% CI -0.44 to -0.14; *P* = .0002; I^2^ = 33%) and 6 months (SMD -0.33; 95% CI -0.51 to -0.15; *P* = .0003; I^2^ = 0%). Compared with tumor necrosis factor inhibitors or placebo, IL-6 inhibition showed greater Lp(a) reductions at 2–3 months (SMD -0.49; 95% CI -0.73 to -0.24; *P* = .0001; I^2^ = 63%) and 6 months (SMD ­-0.97; 95% CI -1.16 to -0.77; *P* < .00001; I^2^ = 0%).

**Conclusions:**

This meta-analysis demonstrates significant reductions in Lp(a) levels with anti-IL­-6/IL-6R therapy vs controls. The relevance of these findings warrants investigation in cardiovascular outcome trials.

**Figure:**
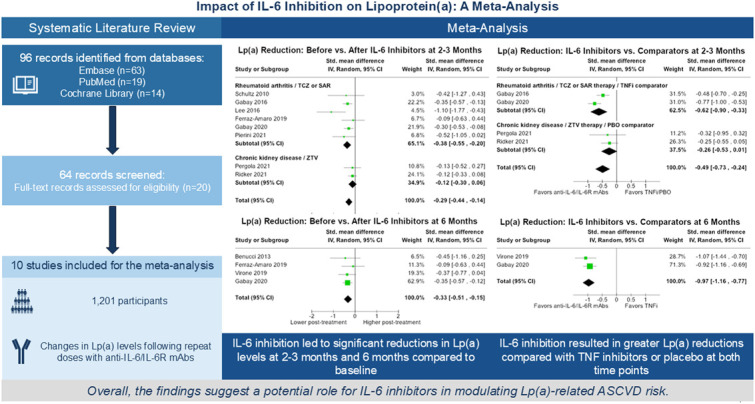
Central Illustration. Systematic review and meta-analysis of IL-6 inhibition and lipoprotein(a) [Lp(a)]. Among 10 studies (1,201 participants), IL-6 inhibitor therapy was associated with reductions in Lp(a) levels at 2-3 months and 6 months compared with baseline and with TNF inhibitors or placebo. These findings suggest that modulation of the IL-6 pathway may affect Lp(a) levels.

## 1 Introduction

Atherosclerotic cardiovascular disease (ASCVD) remains the leading cause of morbidity and mortality worldwide, despite the widespread implementation of lipid-lowering, antihypertensive, and antithrombotic therapies [1]. Even among individuals receiving optimal medical treatment, a substantial burden of cardiovascular events persists, highlighting the challenge of residual cardiovascular risk [2]. Inflammation, as a key driver of atherosclerosis, has emerged as a compelling target for novel therapeutic strategies [3]. Interleukin (IL)-6, which promotes endothelial dysfunction, plaque instability, and thrombosis, has gained attention as a potential modifiable factor in ASCVD progression [4]. This has led to growing interest in IL-6 inhibition as a means of targeting residual inflammation and potentially mitigating cardiovascular risk.

The NLRP3 (NOD-, LRR-, and pyrin domain-containing protein 3) inflammasome and its downstream signaling through IL-1β, IL-6, and C-reactive protein (CRP) play a central role in the pathogenesis of atherosclerosis [5]. Given their contributions to vascular inflammation and plaque progression, targeting these pathways may offer novel therapeutic strategies for ASCVD. IL-6 is a multifunctional cytokine produced by endothelial and smooth muscle cells, macrophages, and T cells; it features pro-atherogenic effects such as endothelial cell activation, leukocyte recruitment, foam cell formation, and plaque rupture [6]. IL-6 also induces hepatic synthesis of CRP, a protein associated with inflammation, thrombosis, and cardiovascular events [7].

Synthesis of highly atherogenic lipoprotein(a) [Lp(a)] is also affected by IL-6. Like IL-6, Lp(a) is an independent and causal risk factor for ASCVD, whereby elevated Lp(a) increases ASCVD risk through various mechanisms, including atherogenesis, inflammation, thrombosis, and arterial calcification [8,9]. Although Lp(a) levels are largely determined by the *LPA* gene, IL-6 can upregulate *LPA* expression via response elements [10,11]. Additionally, IL-6 signaling blockade by monoclonal antibodies (mAb) inhibits apolipoprotein(a) synthesis and is associated with reductions in circulating Lp(a) concentration [12]. As such, IL-6 inhibition has been proposed as a potential treatment mechanism to lower ASCVD risk both directly (i.e., reducing inflammatory risk) and indirectly (i.e., reducing Lp(a) levels) [11,13].

While evidence to date is mixed and varies by population, data from various randomized controlled trials (RCT) evaluating anti-IL-6/IL-6 receptor (IL-6R) treatment for patients with rheumatoid arthritis (RA) or chronic kidney disease (CKD) have reported reductions in Lp(a) levels [11]. Therefore, we conducted a systematic literature review (SLR) and meta-analysis to summarize the clinical evidence for and quantify the effect of anti-IL-6/IL-6R treatment on Lp(a) levels. Methods and key findings are detailed in the graphical abstract. The effects of IL-6 inhibition on concentrations and properties of lipids and lipoproteins other than Lp(a) are detailed in prior reviews [[14], [15], [16], [17], [18]].

## 2 Methods

### 2.1 Search strategy, study selection, and data extraction

The Embase, PubMed and Cochrane Library databases were searched for eligible studies published from any start date to June 30, 2024. Studies were eligible if they reported changes to Lp(a) levels over time following the administration of repeat doses of anti-IL-6/IL-6R mAbs. Search terms included lipoprotein(a), anti-IL-6, and anti-IL-6R, as well as generic names of such antibodies: clazakizumab, olokizumab, sarilumab, satralizumab, siltuximab, sirukumab, tocilizumab, and ziltivekimab (**Supplementary Table 1**). The following study types were excluded from this analysis: non-clinical studies, duplicate studies or those that leveraged the same data as a study already included in this analysis, studies where treatment was administered as a single dose, as well as any studies that did not investigate a treatment of interest or report outcomes of interest.

Search results were exported to Microsoft Excel. Following the manual removal of duplicate records, titles and abstracts were screened for potential relevance by two independent reviewers. Full-text articles of the remaining records were evaluated by both reviewers to further assess their eligibility. Of the studies selected for inclusion in the meta-analysis, the following data were extracted: details of the study (i.e., study design, sample size, underlying disease of enrolled patients, treatments, and dosage); patient baseline characteristics (i.e., age, sex, race/ethnicity, statin use, CRP levels); Lp(a) levels at baseline and following treatment; and the calculated absolute change in Lp(a) levels. Data on changes in high-sensitivity CRP (hs-CRP), apolipoprotein B (ApoB), low-density lipoprotein cholesterol (LDL-C), and high-density lipoprotein cholesterol (HDL-C) were also extracted from studies that reported these outcomes, acknowledging variability in outcome reporting across studies. Institutional Review Board approval was not required for this study as the meta-analysis leveraged already published data and did not involve collecting any new data from human participants.

### 2.2 Statistical analysis

All statistical analyses were performed using the web-based Review Manager software (RevMan Web, Version 8.0.0, The Cochrane Collaboration). *P*-values < 0.05 were considered statistically significant.

The primary analysis employed a random-effects meta-analysis to compare the effect of IL-6 inhibitors on Lp(a) levels vs the comparator group in studies that included both arms. We analyzed the treatment effects at two time points: 2 to 3 months, and 6 months. The secondary pre-post analysis assessed the effect of IL­6 inhibitors on Lp(a) levels before and after the intervention, at 2 to 3 months, and at 6 months; this analysis is reported in the results before the primary analysis. A subgroup analysis also examined these effects by disease type, IL-6 inhibitor, and comparator.

Treatment effects were summarized using standardized mean differences (SMD) and 95 % confidence intervals (95 % CI) to allow pooling across studies reporting the biomarkers in different units. SMD values of 0.2 to 0.5, 0.5 to 0.8, and > 0.8 were considered small, medium, and large, respectively [19]. To enhance clinical interpretability, SMD values were back-transformed into absolute changes using the pooled baseline standard deviations (SD) from the included studies. Additionally, SMDs were converted to percentiles using the standard normal distribution to estimate the proportion of control participants that a typical treated participant would outperform [20]. For Lp(a), separate calculations were performed for nmol/L and mg/dL to minimize inaccuracies related to unit conversion, given the absence of a reliable universal conversion factor. For LDL-C and HDL-C, values reported in mmol/L were converted to mg/dL by multiplying by 38.67.

Studies reporting medians and IQR were converted to means and SD using methods described by Wan et al [21]. If the SE was given, the SD was estimated by SD = SE × √n, where n is the number of subjects [22]. If SDs of absolute changes were missing, they were calculated by using correlation coefficients based on methods proposed by the Cochrane Handbook for Systematic Reviews of Interventions [23]. When correlation coefficients were unavailable, they were imputed from other studies included in the analysis that had similar treatment and follow-up periods; when no comparable studies were available, a correlation of 0.5 was assumed [23]. In cases where the imputed correlation yielded no valid solution due to mathematical constraints, follow-up SDs were assumed equal to baseline SDs [23]. For studies with multiple treatment arms of the same agent, all arms were combined using formulas from the Cochrane Handbook [24].

Heterogeneity was quantified using the I^2^ statistic, with the following interpretation: 0 % to 40 % might not be important; 30 % to 60 % may represent moderate heterogeneity; 50 % to 90 % may represent substantial heterogeneity; and 75 % to 100 % indicates considerable heterogeneity [21]. Sensitivity analyses were performed using the leave-one-out method, removing one study at a time and repeating the analysis for all studies. Funnel plots (including Egger’s test and trim-and-fill tests) were not used to assess publication bias because all analyses included fewer than 10 papers, as per Cochrane guidance [25].

## 3 Results

### 3.1 Summary of included studies

**Fig. 1:**
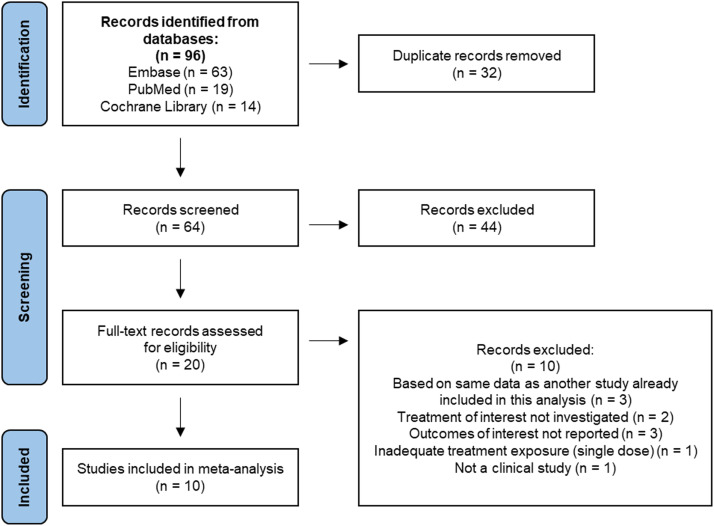
Preferred Reporting Items for Systematic Reviews and Meta-Analyses (PRISMA) Flow Diagram.

Out of 96 records retrieved across the three databases, 32 duplicates were removed. An additional 44 and 10 records were removed after reviewing titles/abstracts and full-text publications, respectively (Fig. 1). In total, the analyses included the following 10 studies (2010 to 2021), representing 1,201 patients who received either an anti-IL-6/IL-6R mAb (n = 711) or a comparator (n = 490): six RCTs [[26], [27], [28], [29], [30], [31]], three prospective observational studies [18,32,33], and one prospective interventional study [34] (Table 1). The most common IL­-6/IL-­6R antibody evaluated in the selected studies was tocilizumab (n = 7), followed by ziltivekimab (n = 2) and sarilumab (n = 1). Clazakizumab, which was evaluated in the Phase 2b POSIBIL_6_ESKD study, was not included in this meta-analysis as the necessary data were unavailable before study assessments and manuscript development.

**Table 1:** Summary of Included Studies and Baseline Characteristics^a^.

Study	Study type	Number of patients	Age, years^b^	Female sex, %	Underlying condition	IL-6/IL-6R antibody	Comparator	CRP (mg/L)^b^	Follow-up^d^	Lipoprotein(a)^b^
Gabay 2016 [26] (ADACTA)	RCT	324	53–54	80–82	Rheumatoid arthritis	Tocilizumab	Adalimumab	25–26	8 weeks	22–26 mg/dL
Lee 2016 [28] (MEASURE)	RCT	20	59^c^	100	Rheumatoid arthritis	Tocilizumab	Placebo	12.8	12 weeks	30 mg/dL
Virone 2019 [31] (ROC)	RCT	143	57^c^	82	Rheumatoid arthritis	Tocilizumab	TNF inhibitor	8.5^c^	24 weeks	10–15 mg/dL^c^
Gabay 2020 [27] (MONARCH)	RCT	307	50–53	79–84	Rheumatoid arthritis	Sarilumab	Adalimumab	17.4–23.6	12, 24 weeks	17.9–23.6 mg/dL^c^
Ridker 2021 [30] (RESCUE)	RCT	264	66–70.0^c^	44–55	Non–dialysis-dependent CKD	Ziltivekimab	Placebo	5.5–5.8^c^	12 weeks	37–50 nmol/L^c^
Pergola 2021 [29]	RCT	61	58–64	25–56	Hemodialysis-dependent CKD, anemia	Ziltivekimab	Placebo	4.0–13.2^c^	12 weeks	22–64 nmol/L^c^
Ferraz-Amaro 2019 [34]	Prospective interventional	27	52	88	Rheumatoid arthritis	Tocilizumab	None	88^c^	3, 6, 12 months	29 mg/dL
Schultz 2010 [33]	Prospective observational	11	51	64	Rheumatoid arthritis	Tocilizumab	None	–	1, 3 months	35 mg/dL
Benucci 2013 [32]	Prospective observational	16	56	100	Rheumatoid arthritis	Tocilizumab	None	38	6 months	28 mg/dL
Pierini 2021 [18]	Prospective observational	28	61	89	Rheumatoid arthritis	Tocilizumab	None	3.9^c^	3 months	48 mg/dL^c^

CKD, chronic kidney disease; CRP, C-reactive protein; IL-6, interleukin-6; IL-6R, interleukin-6 receptor; RCT, randomized controlled trial; TNF, tumor necrosis factor.

a Ranges are shown for studies that did not report baseline data for the total population.

b Values are shown as means unless noted otherwise.

c Median value.

d For lipoprotein(a) measurement.

The mean age across the study groups ranged from 50.4 to 70.0 years, and the included studies enrolled between 25 % and 100 % female patients. Eight of the 10 studies enrolled patients with RA, and the remaining two enrolled patients with CKD. The follow-up duration ranged from 2 to 12 months. The average baseline Lp(a) levels across all studies and treatment groups were 65.7 (SD 89.5) nmol/L among 325 participants and 25.7 (SD 32.8) mg/dL among 876 participants.

### 3.2 IL-6 inhibitors and Lp(a) levels

**Fig. 2:**
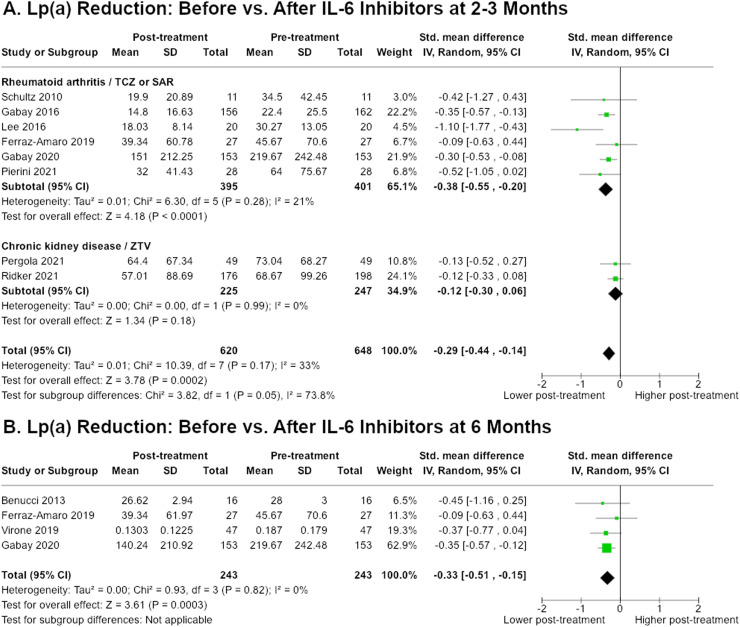
Reduction in Lipoprotein(a) Before vs After Treatment With an Anti-IL-6/IL-6 Receptor Monoclonal Antibody at (A) 2 to 3 Months and (B) 6 Months. *CI, confidence interval; IL-6, interleukin-6; IV, inverse variance; Lp(a), lipoprotein(*a*); SAR, sarilumab; SD, standard deviation; Std, standardized; TCZ, tocilizumab, ZTV, ziltivekimab.*

Eight studies (n = 648) [18,[26], [27], [28], [29], [30],^33^,^34^] provided the data necessary to compare pre- and post-treatment Lp(a) levels at 2 to 3 months following anti-IL-6/IL-6R mAb therapy in the secondary analysis. Pooled analysis indicated small but statistically significant reductions in Lp(a) at 2 to 3 months with SMD of -0.29 (95 % CI -0.44 to -0.14) (Fig. 2**A**). When back-transformed using the baseline Lp(a) SD across studies, this translated to a Lp(a) reduction of 25.9 nmol/L or 9.5 mg/dL. Furthermore, this SMD corresponded to the 38.6th percentile, meaning that the average participant’s post-treatment Lp(a) levels were lower than 61.4 % of the pre-treatment levels. The findings remained significant on leave-one-out sensitivity analysis, indicating robust results. There was negligible heterogeneity with an I^2^ of 33 % (P = .17), driven mainly by Lee 2016 [28].

Subgroup analyses were also performed by population type and anti-IL-6/IL-6R mAb. In six studies [18,[26], [27], [28],^33^,^34^], participants with RA (n = 401) who were treated with tocilizumab or sarilumab showed an SMD of -0.38 (95 % CI -0.55 to -0.20; *P* < .0001), which translated to an Lp(a) reduction of 34.0 nmol/L or 12.4 mg/dL (Fig. 2**A**). This SMD corresponded to the 35.2nd percentile. Furthermore, the findings remained significant on leave-one-out sensitivity analysis. In two studies [29,30], participants with CKD (n = 247) who were treated with ziltivekimab showed an SMD of ­-0.12 (95 % CI -0.30 to 0.06; *P* = .18); the lack of statistical significance indicated no major Lp(a)­-lowering in this population (Fig. 2**A**). Upon direct comparison, the difference in Lp(a) reduction between subgroups was statistically significant (*P* = .05).

Four studies with 6-month follow-up data were pooled (n = 243) [27,31,32,34]. All studies included participants with RA who were treated with tocilizumab or sarilumab. Pooled analysis indicated small but statistically significant reductions in Lp(a) at 6 months with SMD of ­-0.33 (95 % CI ­-0.51 to -0.15; *P* = .0003), which translated to a reduction of 29.5 nmol/L or 10.8 mg/dL (Fig. 2**B**). This SMD corresponded to the 37.1st percentile, meaning that the average participant in the treatment group had Lp(a) levels lower than 62.9 % of participants in the control group. There was no heterogeneity (I^2^ = 0 %). Lastly, one study (Ferraz-Amaro [34]) reported Lp(a) levels at 12 months with an absolute reduction of -6 mg/dL (IQR -33 to -0).

### 3.3 Comparative Lp(a) response: IL-6 inhibitors vs comparators

**Fig. 3:**
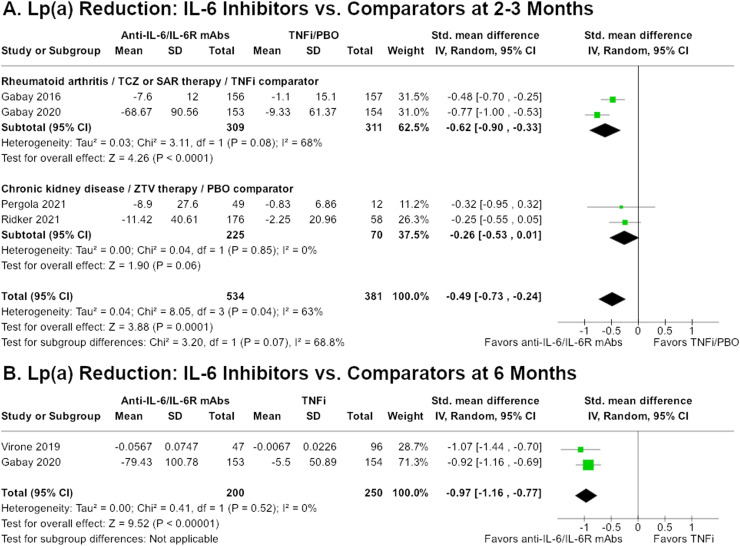
Reduction in Lipoprotein(a) Following Treatment With an Anti-IL-6/IL-6 Receptor Monoclonal Antibody vs a Comparator at (A) 2 to 3 Months and (B) 6 Months. CI, confidence interval; IL-6, interleukin-6; IL-6R, interleukin-6 receptor; IV, inverse variance; Lp(a), lipoprotein(a); mAb, monoclonal antibody; PBO, placebo; SAR, sarilumab; SD, standard deviation; Std, standardized; TCZ, tocilizumab; TNFi, tumor necrosis factor inhibitor; ZTV, ziltivekimab.

Four studies (n = 915) that reported 2–3 months’ follow-up data were pooled in the primary analysis: 534 participants received anti-IL-6/IL-6R mAb therapy, and 381 received a comparator [26,27,29,30]. Pooled analysis indicated a small-to-moderate reduction in Lp(a) at 2 to 3 months with an SMD of ­-0.49 (95 % CI -0.73 to -0.24; *P* = .0001) (Fig. 3**A**). When back-transformed using the baseline Lp(a) SD across studies, this translated to a reduction of 43.8 nmol/L or 16.0 mg/dL. This SMD corresponded to the 31.2nd percentile, meaning that the average participant in the treatment group had Lp(a) levels lower than 68.8 % of participants in the control group. The findings remained significant on leave-one-out sensitivity analysis, indicating robust results. There was substantial heterogeneity with an I^2^ of 63 % (*P* = .04), primarily driven by Gabay 2020 [27].

Subgroup analyses were also performed by population type, anti-IL-6/IL-6R mAb, and comparator. Two studies included participants with RA (n = 620) who were treated with tocilizumab, sarilumab, or a comparator (tumor necrosis factor inhibitor [TNFi]) [26,27]. These participants showed an SMD of -0.62 (95 % CI -0.90 to -0.33; *P* < .0001), which translated to a Lp(a) reduction of 55.5 nmol/L or 20.3 mg/dL (Fig. 3**A**). This SMD corresponded to the 26.8th percentile. Two studies included participants with CKD (n = 295) who were treated with ziltivekimab or a placebo comparator [29,30]. Participants showed an SMD of -0.26 (95 % CI -0.53 to 0.01; *P* = .06), indicating small reductions in Lp(a) levels with ziltivekimab treatment that did not reach statistical significance compared with placebo treatment (Fig. 3**A**). The subgroups did not differ significantly upon direct comparison (*P* = .07).

Two studies (n = 450) reported 6-month follow-up data, which were pooled: 200 participants with RA who received tocilizumab [31] or sarilumab [27], and 250 who received a comparator (TNFi) [27,31]. Pooled analysis indicated a large reduction in Lp(a) at 6 months with an SMD of -0.97 (95 % CI -1.16 to -0.77; *P* < .00001), which translated to a reduction of 86.8 nmol/L or 31.8 mg/dL (Fig. 3**B**). This SMD corresponded to the 16.6th percentile, meaning that the average participant in the treatment group had Lp(a) levels lower than 83.4 % of participants in the control group. There was no heterogeneity (I^2^ = 0 %).

### 3.4 Effects on hs-CRP, apolipoprotein B, LDL-C, and HDL-C

To characterize the broader effects of IL-6 pathway inhibition on inflammatory and lipid markers, secondary analyses examined changes in hs-CRP, ApoB, LDL-C, and HDL-C (**Figures S1–S4**). The average baseline hs-CRP level across all studies and treatment groups among 392 participants [18,[28], [29], [30],^34^] was 0.73 (SD 0.76) mg/dL. Pre- versus post-treatment analyses, which compared values before and after anti-IL-6/IL-6R mAb therapy within treatment groups, demonstrated large reductions in hs-CRP at 3 months (SMD -1.28; 95 % CI -1.75 to -0.81; *P* < .00001), corresponding to a reduction of 0.98 mg/dL, with significant reductions in both RA and CKD subgroups (**Figure S1A**). In the two CKD studies that compared anti-IL-6 mAb therapy versus placebo [29,30], hs-CRP was similarly reduced (SMD -1.33; 95 % CI -1.97 to -0.70; *P* < .0001), corresponding to a reduction of 1.01 mg/dL (**Figure S1B**).

The average baseline ApoB level across all studies and treatment groups among 482 participants [18,28,30,31,34] was 93.3 (SD 29.7) mg/dL. Despite reductions in systemic inflammation, anti-IL-6/IL-6R mAb therapy was associated with small increases in ApoB. Pre- versus post-treatment analyses showed an increase at 3 months (SMD 0.42; 95 % CI 0.02 to 0.82; *P* = .04), corresponding to an increase of 12.5 mg/dL, but not at 6 months (SMD 0.16; 95 % CI -0.16 to 0.48; *P* = .33), with neither RA nor CKD subgroups individually significant at either timepoint (**Figure S2A–B**). The analysis comparing anti-IL-6/IL-6R mAbs versus TNFi or placebo at 3 to 6 months showed a similar pattern (SMD 0.28; 95 % CI 0.06 to 0.51; *P* = .01), corresponding to an increase of 8.3 mg/dL, with neither subgroup individually significant (**Figure S2C**).

The average baseline LDL-C level across all studies and treatment groups among 700 participants [18,26,[28], [29], [30],[32], [33], [34]] was 99.4 (SD 37.1) mg/dL. LDL-C showed small increases in pre- versus post-treatment analyses at 2 to 3 months (SMD 0.41; 95 % CI 0.11 to 0.70; *P* = .008), corresponding to an increase of 15.2 mg/dL, but not at 6 months (SMD 0.16; 95 % CI -0.26 to 0.59; *P* = .45) (**Figure S3A–B**). These increases were driven by the RA subgroup (*P* < .00001 for subgroup differences), with no significant changes in patients with CKD treated with ziltivekimab (**Figure S3A**). A similar pattern was observed in the analysis versus TNFi or placebo at 2 to 3 months (SMD 0.40; 95 % CI 0.03 to 0.76; *P* = .03), corresponding to an increase of 14.8 mg/dL, with significant increases in the RA/tocilizumab subgroup but not the CKD/ziltivekimab subgroup (*P* = .01 for subgroup differences) (**Figure S3C**).

The average baseline HDL-C level across all studies and treatment groups among 713 participants [18,26,[28], [29], [30],[32], [33], [34]] was 52.1 (SD 16.9) mg/dL. HDL-C showed small increases in pre- versus post-treatment analyses at 2 to 3 months (SMD 0.30; 95 % CI 0.11 to 0.48; *P* = .002), corresponding to an increase of 5.1 mg/dL, but not at 6 months (SMD 0.32; 95 % CI -0.50 to 1.15; *P* = .44), with significant increases in the RA but not CKD subgroup (**Figure S4A–B**). In the analysis versus TNFi or placebo at 2 to 3 months, HDL-C showed small increases (SMD 0.43; 95 % CI 0.16 to 0.70; *P* = .002), corresponding to an increase of 7.3 mg/dL, with significant increases in both RA and CKD subgroups (**Figure S4C**).

## 4 Discussion

This meta-analysis identified significant reductions in Lp(a) levels following IL-6/IL-6R inhibition. Pooled analyses demonstrated small but significant Lp(a) reductions at 2 to 3 months and 6 months compared with baseline. Compared with TNFi or placebo, IL-6 inhibition resulted in greater reductions, with the effect size increasing over time. Although not included in this analysis, results from the Phase 2b POSIBIL_6_ESKD study of 127 hemodialysis patients also demonstrated significant reductions in Lp(a) of 38 %, 37 %, and 52 % from baseline median Lp(a) levels of 56 to 77 nmol/L with clazakizumab 2.5, 5, and 10 mg IV monthly, respectively [35].

Among its hypothesized atherothrombotic effects, IL-6 stimulates hepatic synthesis of acute-phase reactants including fibrinogen, plasminogen activator inhibitor-1, and apolipoprotein(a) [11,36,37]. Apolipoprotein(a) expression is biologically linked to IL-6 due to the presence of an IL-6 response element on the LPA gene [12,38]. Consistent with this biological link, in vitro and epidemiological studies have demonstrated an association between levels of IL-6 and Lp(a). Transcriptomic analysis of human liver biopsies showed a correlation between gene expression of *LPA* and IL-6 response genes, and tocilizumab, an anti-IL-6R monoclonal antibody, inhibited IL-6-induced *LPA* expression by human hepatocytes [12]. In a cross-sectional study in Germany (N = 1,153), levels of Lp(a) were significantly positively correlated with IL-6 levels (*P* = .0005) [12]. These findings supported the current meta-analysis, confirming a reduction in Lp(a) with IL-6 pathway inhibition.

The clinical relevance of the observed Lp(a)-lowering with anti-IL-6/IL-6R monoclonal antibodies remains unknown. Analysis of the alirocumab Phase 3 ODYSSEY study showed that each 5 mg/dL reduction in Lp(a) predicted a 2.5 % reduction in major adverse cardiovascular events [39]. Human genetic studies have predicted that Lp(a)-lowering by 50 to 100 mg/dL may be needed to reduce cardiovascular risk by 20 % [40,41].

### 4.1 Limitations

This meta-analysis has several limitations that should be considered when interpreting the results. Firstly, one limitation of this meta-analysis is the limited number of available studies and the low number of participants in most studies. Despite a comprehensive search strategy, only 64 unique publications were identified prior to screening, indicating that this research area remains relatively underexplored. Consequently, the pooled estimates and I^2^ heterogeneity statistics should be interpreted with caution. I^2^ estimates are imprecise when based on fewer than 10 studies, but we reported I^2^ values alongside sensitivity analyses to provide transparency into between-study variability.

Secondly, the skewed distribution of Lp(a) may affect the accuracy of the pooled estimates. In addition, significant heterogeneity in Lp(a) reporting across studies (e.g., use of nmol/L or mg/dL) necessitated using SMD for analyses. Although this approach accounts for measurement differences, it limits direct clinical interpretation as SMD is a unitless measure. To address this, SMD was back­transformed to absolute Lp(a) values; however, these measures should be interpreted cautiously. These transformations used pooled SD across all studies, combining participants with RA and CKD, and were employed in analyses that merged data reported in different units from different populations. This heterogeneity may affect the accuracy and generalizability of back-transformed values to specific patient groups or clinical contexts.

Thirdly, percent changes in Lp(a) levels could not be pooled due to inconsistent reporting and missing data, potentially limiting the evaluation of relative changes. Analysis of comparator groups was restricted by insufficient data, particularly regarding SD, which prevented comprehensive pre- and post-treatment comparisons. Some studies were also excluded due to incomplete data reporting or limited data availability at the time of this analysis, such as the Phase 2b clazakizumab trial, which demonstrated larger reductions in Lp(a) than other included studies. In some cases, SD was imputed based on values from other studies, which introduced additional uncertainty. Furthermore, publication bias could not be formally assessed due to the small number of included studies; therefore, the possibility of selective reporting remains a concern. These limitations underscore the need for standardized Lp(a) data reporting in future studies, including the consistent use of measurement units and comprehensive reporting of statistical parameters to facilitate more robust and clinically interpretable meta-analyses.

Furthermore, although the clinically relevant population for this meta-analysis comprises those with high Lp(a) at baseline, such patients were not enrolled in the included studies. Such studies are needed to more accurately ascertain the magnitude of Lp(a) reduction with IL-6 inhibition. Lastly, the lower Lp(a) levels observed in these studies may actually reflect increases from baseline driven by patients’ underlying conditions, with IL-6 pathway blockade merely restoring Lp(a) to baseline levels. In this case, IL-6 inhibition may not have an effect in the absence of severe inflammatory states, which may limit its effectiveness in patients with mild elevations in hs-CRP levels.

## 5 Conclusions

Overall, this meta-analysis demonstrates that IL-6 inhibition leads to modest but significant reductions in Lp(a) levels compared with controls. Given the well-established independent roles of both inflammation and Lp(a) in ASCVD, these findings suggest that IL-6 blockade may offer a dual benefit by targeting both pathways. Whether this observed magnitude of Lp(a) reduction translates into meaningful cardiovascular risk reduction remains unknown. Results from ongoing RCTs investigating anti-IL-6/IL-6R therapies are eagerly awaited to characterize the potential benefits and risks of IL-6 inhibition in high-risk patients with cardiovascular disease (Table 2). Additional studies may be particularly informative.

**Table 2:** Ongoing Randomized Controlled Trials Investigating Anti-IL-6/IL-6R Therapy for the Treatment of Patients With or at High Risk of Cardiovascular Disease.

Antibody	Study	NCT number	Phase	Underlying disease	Comparator	Primary completion (estimated)	Study completion (estimated)	Enrollment (estimated)	Primary endpoint(s)
Ziltivekimab	ZEUS	NCT05021835	3	ASCVD, CKD, elevated hs-CRP	Placebo	September 2025	January 2026	6200	Time to first occurrence of 3-point MACE
Ziltivekimab	ARTEMIS	NCT06118281	3	AMI	Placebo	September 2026	September 2026	10,000	Time to first occurrence of 3-point MACE
Ziltivekimab	HERMES	NCT05636176	3	HFpEF, elevated hs-CRP	Placebo	July 2027	July 2027	5600	Time to first occurrence of CV death, HF hospitalization, or urgent HF visit
Ziltivekimab	ATHENA	NCT06200207	3	HFpEF, elevated hs-CRP	Placebo	May 2026	August 2026	680	Change in KCCQ-CSS
Clazakizumab	POSIBIL_6_ESKD	NCT05485961	2b/3	ASCVD or diabetes mellitus, hemodialysis-dependent ESKD	Placebo	December 2028	December 2028	2310	Change from baseline in CRP (Phase 2b) Time to first occurrence of CV death or MI (Phase 3)
Pacibekitug (TOUR006)	TRANQUILITY	NCT06362759	2	CKD, elevated hs-CRP	Placebo	May 2025	February 2026	143 (actual)	Change from baseline in CRP

AMI, acute myocardial infarction; ASCVD, atherosclerotic cardiovascular disease; CKD, chronic kidney disease; CSS, clinical summary score; CV, cardiovascular; ESKD, end-stage kidney disease; HFpEF, heart failure with preserved ejection fraction; hs-CRP, high-sensitivity C-reactive protein; KCCQ, Kansas City Cardiomyopathy Questionnaire; MACE, major adverse cardiovascular event; MI, myocardial infarction; NCT, National Clinical Trial

## Data availability

The data that support the findings of this meta-analysis are available from the individual studies included. Additional details on the included studies can be made available by the corresponding author.

## Funding

This research did not receive any specific grant from funding agencies in the public, commercial, or not-for-profit sectors.

## Declaration of competing interest

SM is supported by the National Heart, Lung, and Blood Institute of the National Institutes of Health (T32-HL-076132). JM and MIA declare no competing interests. JW is an employee of Tourmaline Bio, Inc, and EdG was an employee of Tourmaline Bio, Inc at the time of research and manuscript development. MDS is supported by institutional grants from Amgen, Arrowhead, Boehringer Ingelheim, 89Bio, Esperion, Novartis, Ionis, Merck, New Amsterdam, and Cleerly; has participated in Scientific Advisory Boards with Amgen, Ionis, Novartis, New Amsterdam, and Merck; and has served as a consultant for Ionis, Novartis, Regeneron, Aidoc, Kaneka, Novo Nordisk, Arrowhead, and Tourmaline. Given his role as Associate Editor on the Editorial Board of the American Journal of Preventive Cardiology, MDS had no involvement in the peer-review of this article and has no access to information regarding its peer-review. Full responsibility for the editorial process for this article was delegated to another journal editor.

## CRediT authorship contribution statement

**Saeid Mirzai:** Writing – review & editing, Writing – original draft, Methodology, Conceptualization. **James McParland:** Writing – review & editing, Writing – original draft. **Muhammad Imtiaz Ahmad:** Writing – review & editing, Writing – original draft. **Emil deGoma:** Writing – review & editing, Writing – original draft, Methodology, Conceptualization. **John Walsh:** Writing – review & editing, Writing – original draft, Methodology, Conceptualization. **Michael D. Shapiro:** Writing – review & editing, Writing – original draft, Methodology, Conceptualization.

## Declaration of competing interest

The authors declare the following financial interests/personal relationships which may be considered as potential competing interests: Saeid Mirzai reports writing assistance was provided by Tourmaline Bio, Inc., and a relationship with the National Heart, Lung, and Blood Institute of the National Institutes of Health that includes: funding or grants. James McParland writing assistance was provided by Tourmaline Bio, Inc. There are no additional relationships to disclose. Muhammad Imtiaz Ahmad writing assistance was provided by Tourmaline Bio, Inc. There are no additional relationships to disclose. Emil deGoma reports writing assistance was provided by Tourmaline Bio, Inc., and a relationship with Tourmaline Bio, Inc., that includes: employment. John Walsh reports writing assistance was provided by Tourmaline Bio, Inc., and a relationship with Tourmaline Bio, Inc., that includes: employment. Michael D. Shapiro reports writing assistance was provided by Tourmaline Bio, Inc.; a relationship with Amgen, Arrowhead, Boehringer Ingelheim, 89Bio, Esperion, Novartis, Ionis, Merck, New Amsterdam, and Cleerly that includes: funding or grants; a relationship with Amgen, Ionis, Novartis, New Amsterdam, Merck, Regeneron, Aidoc, Kaneka, Novo Nordisk, Arrowhead, and Tourmaline that includes: consultation or advisory fees; and a relationship with the American Journal of Preventive Cardiology that includes: editorial board member. Given his role as Associate Editor on the Editorial Board of the American Journal of Preventive Cardiology, Michael D. Shapiro had no involvement in the peer-review of this article and has no access to information regarding its peer-review. Full responsibility for the editorial process for this article was delegated to another journal editor.

## Appendix Supplementary materials

Image, application 1

Image, application 2
