# Supplementary material for: Effect of IL-6 inhibition on lipoprotein(a) levels: A systematic review and meta-analysis
Source: Am J Prev Cardiol. 2026 Feb 22;27:101498. doi: 10.1016/j.ajpc.2026.101498 (PMC13261205; doi:10.1016/j.ajpc.2026.101498)
Supplement: Supplementary file 1 [file mmc1.docx]

**Supplementary Figure Legends**

**Supplementary Figure 1.** Change in hs-CRP levels (A) before vs after treatment with an anti-IL-6/IL-6 receptor monoclonal antibody at 3 months and (B) following treatment with an anti-IL-6/IL-6 receptor monoclonal antibody vs placebo at 3 months.


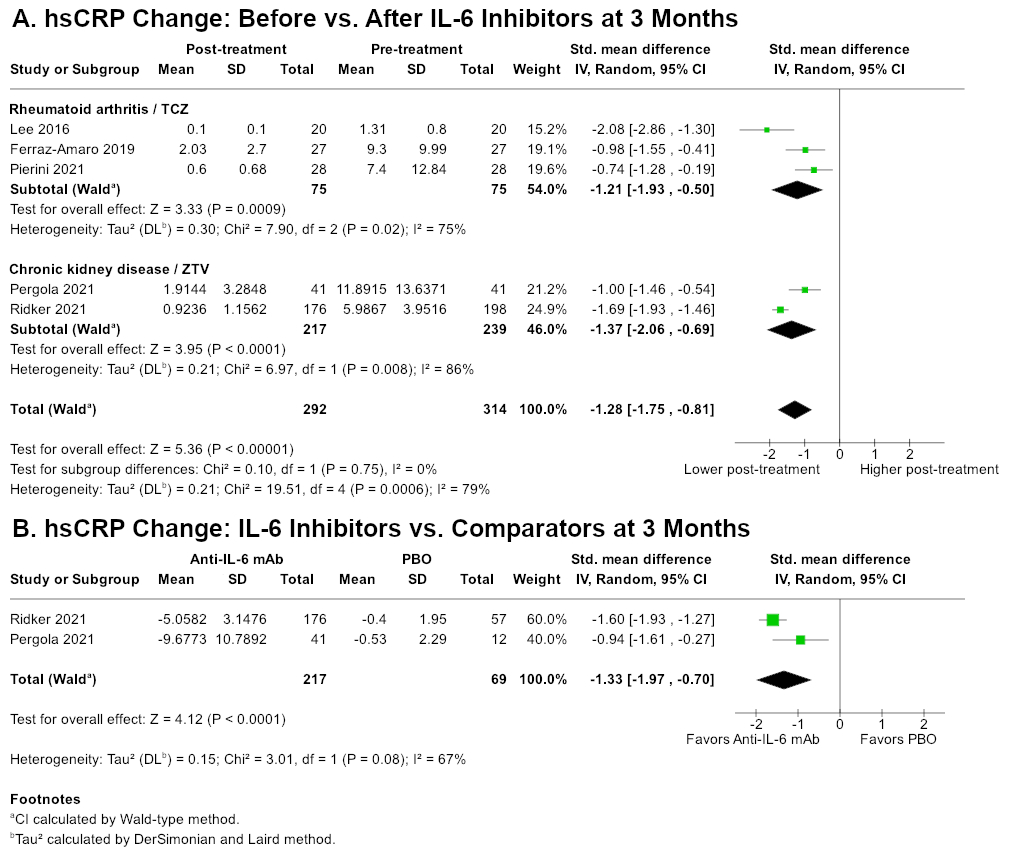


^a^CI calculated by Wald-type method.

^b^Tau^2^ calculated by DerSimonian and Laird method.

CI, confidence interval; hs-CRP, high-sensitivity C-reactive protein; IL-6, interleukin-6; IV, inverse variance; mAb, monoclonal antibody; PBO, placebo; SD, standard deviation; Std, standardized; TCZ, tocilizumab; ZTV, ziltivekimab.

**Supplementary Figure 2.** Change in ApoB levels before vs after treatment with an anti-IL-6/IL-6 receptor monoclonal antibody at (A) 3 months and (B) 6 months; and (C) following treatment with an anti-IL-6/IL-6 receptor monoclonal antibody vs a comparator at 3-6 months.


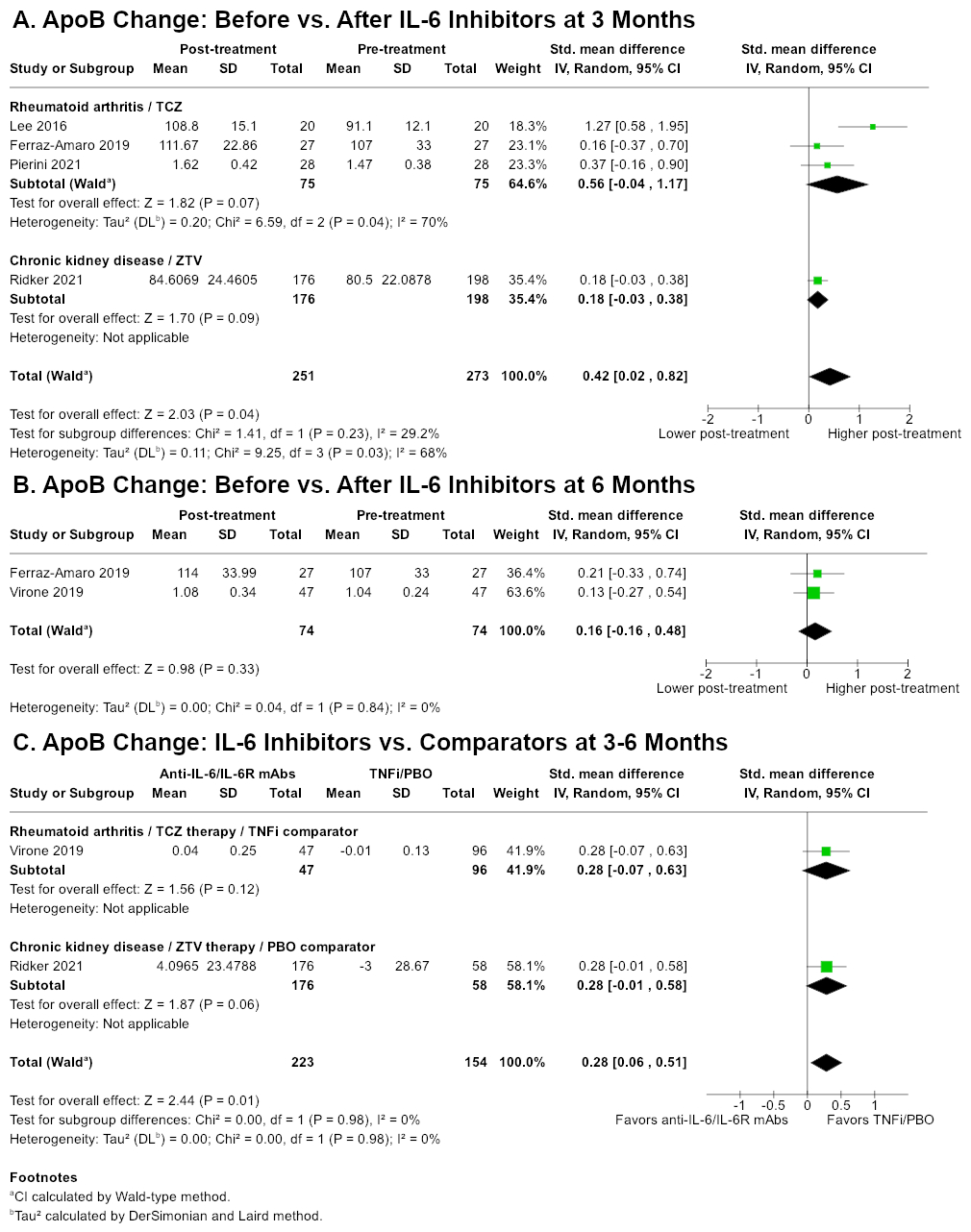


^a^CI calculated by Wald-type method.

^b^Tau^2^ calculated by DerSimonian and Laird method.

ApoB, apolipoprotein B; CI, confidence interval; IL-6, interleukin-6; IL-6R, interleukin-6 receptor; IV, inverse variance; mAb, monoclonal antibody; PBO, placebo; SD, standard deviation; Std, standardized; TCZ, tocilizumab; TNFi, tumor necrosis factor inhibitor; ZTV, ziltivekimab.

**Supplementary Figure 3.** Change in LDL-C levels before vs after treatment with an anti-IL-6/IL-6 receptor monoclonal antibody at (A) 2-3 months and (B) 6 months; and (C) following treatment with an anti-IL-6/IL-6 receptor monoclonal antibody vs a comparator at 2-3 months.


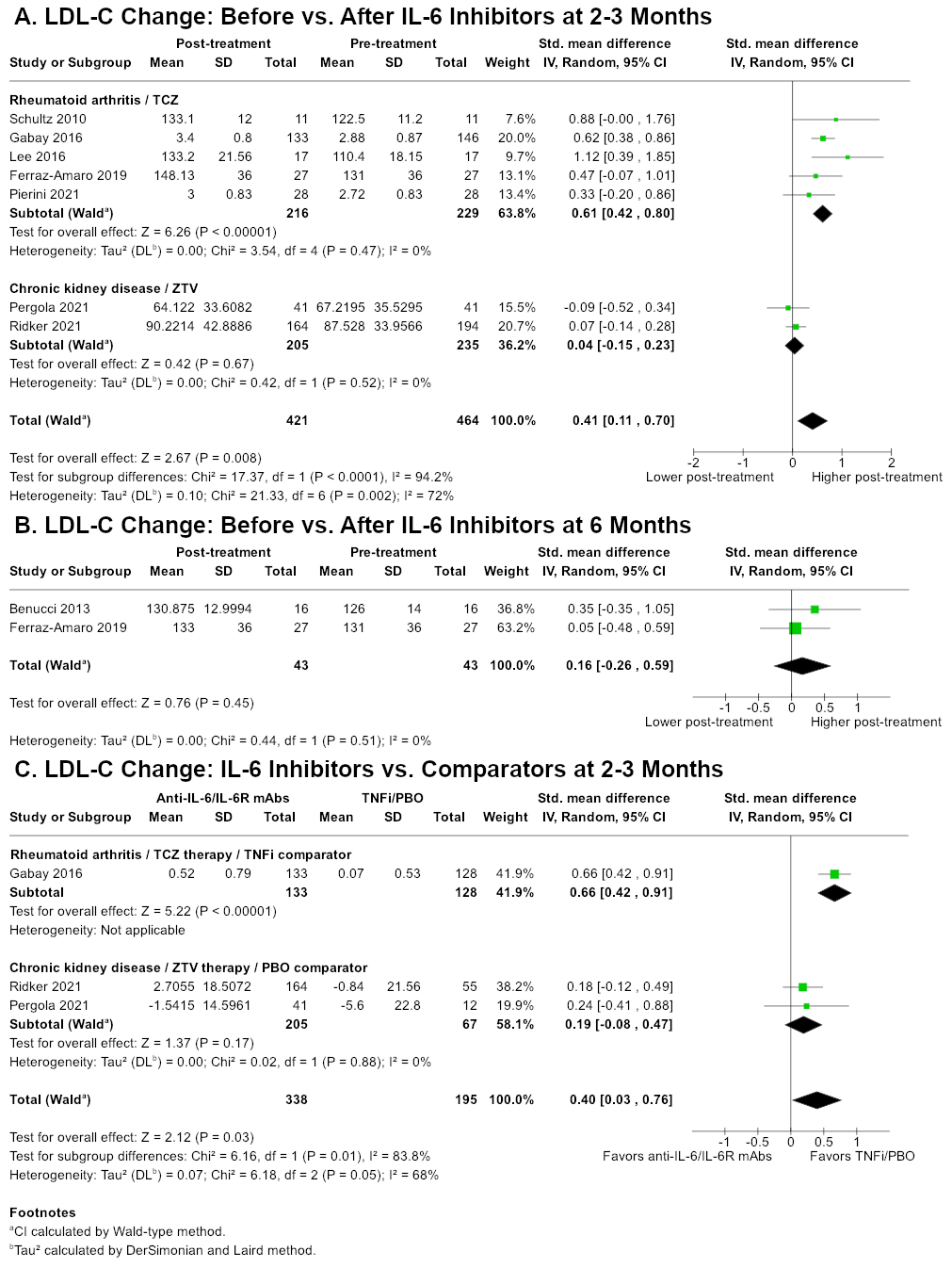


^a^CI calculated by Wald-type method.

^b^Tau^2^ calculated by DerSimonian and Laird method.

CI, confidence interval; IL-6, interleukin-6; IL-6R, interleukin-6 receptor; IV, inverse variance; LDL-C, low-density lipoprotein cholesterol; mAb, monoclonal antibody; PBO, placebo; SD, standard deviation; Std, standardized; TCZ, tocilizumab; TNFi, tumor necrosis factor inhibitor; ZTV, ziltivekimab.

**Supplementary Figure 4.** Change in HDL-C levels before vs after treatment with an anti-IL-6/IL-6 receptor monoclonal antibody at (A) 2-3 months and (B) 6 months; and (C) following treatment with an anti-IL-6/IL-6 receptor monoclonal antibody vs a comparator at 2-3 months.


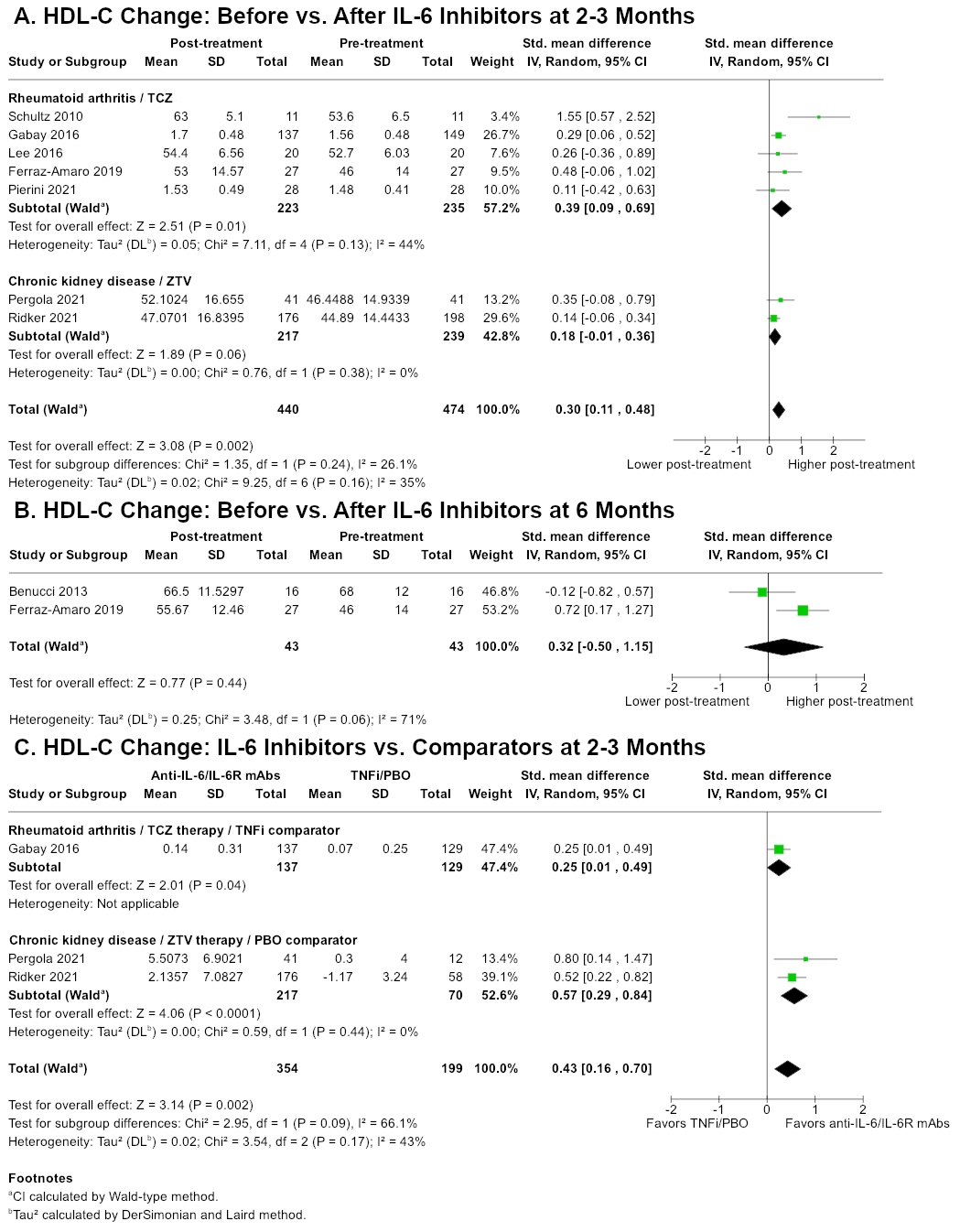


^a^CI calculated by Wald-type method.

^b^Tau^2^ calculated by DerSimonian and Laird method.

CI, confidence interval; HDL-C, high-density lipoprotein cholesterol; IL-6, interleukin-6; IL-6R, interleukin-6 receptor; IV, inverse variance; mAb, monoclonal antibody; PBO, placebo; SD, standard deviation; Std, standardized; TCZ, tocilizumab; TNFi, tumor necrosis factor inhibitor; ZTV, ziltivekimab.
