# Supplementary material for: Effect of IL-6 inhibition on lipoprotein(a) levels: A systematic review and meta-analysis
Source: Am J Prev Cardiol. 2026 Feb 22;27:101498. doi: 10.1016/j.ajpc.2026.101498 (PMC13261205; doi:10.1016/j.ajpc.2026.101498)
Supplement: Supplementary file 2 [file mmc2.docx]

**Supplementary Material**

**Supplementary Table 1.** Search strings used.

| Database | Search String |
| --- | --- |
| Embase | ('lipoprotein(a)' OR 'lp(a)' OR 'lipoprotein (a)' OR 'lp (a)') AND (tocilizumab OR sarilumab OR satralizumab OR siltuximab OR sirukumab OR clazakizumab OR ziltivekimab OR olokizumab OR 'anti-interleukin-6' OR 'anti-il-6' OR 'anti-il-6r' OR (('interleukin-6' OR 'il-6' OR 'il-6r') NEAR/5 antibody)) |
| PubMed | ("lipoprotein(a)" OR "lp(a)" OR "lipoprotein (a)" OR "lp (a)") AND (tocilizumab OR sarilumab OR satralizumab OR siltuximab OR sirukumab OR clazakizumab OR ziltivekimab OR olokizumab OR "anti-interleukin-6" OR "anti-il-6" OR "anti-il-6r" OR "interleukin-6 antibody"[title/abstract:~5] OR "il-6 antibody" [title/abstract:~5] OR "il-6r antibody" [title/abstract:~5]) |
| Cochrane Library | (“lipoprotein(a)” OR “lp(a)” OR “lipoprotein (a)” OR “lp (a)”) AND (tocilizumab OR sarilumab OR satralizumab OR siltuximab OR sirukumab OR clazakizumab OR ziltivekimab OR olokizumab OR “anti-interleukin-6” OR “anti-il-6” OR “anti-il-6r” OR ((“interleukin-6” OR “il-6” OR “il-6r”) NEAR/5 antibody)) |
